# Development of a prediction model for target positioning by using diaphragm waveforms extracted from CBCT projection images

**DOI:** 10.1002/acm2.14112

**Published:** 2023-08-06

**Authors:** Yuta Sakurai, Shintaro Ambo, Mitsuhiro Nakamura, Hiraku Iramina, Yusuke Iizuka, Takamasa Mitsuyoshi, Yukinori Matsuo, Takashi Mizowaki

**Affiliations:** ^1^ Department of Advanced Medical Physics, Graduate School of Medicine Kyoto University Kyoto Japan; ^2^ Department of Radiation Oncology and Image‐Applied Therapy, Graduate School of Medicine Kyoto University Kyoto Japan; ^3^ Department of Radiation Oncology Shizuoka City Shizuoka Hospital Shizuoka Japan; ^4^ Department of Radiation Oncology Kobe City Medical Center General Hospital Hyogo Japan

**Keywords:** Amsterdam Shroud, four‐dimensional cone‐beam computed tomography, prediction accuracy, real‐time tumor tracking

## Abstract

**Purpose:**

To develop a prediction model (PM) for target positioning using diaphragm waveforms extracted from CBCT projection images.

**Methods:**

Nineteen patients with lung cancer underwent orthogonal rotational kV x‐ray imaging lasting 70 s. IR markers placed on their abdominal surfaces and an implanted gold marker located nearest to the tumor were considered as external surrogates and the target, respectively. Four different types of regression‐based PM were trained using surrogate motions and target positions for the first 60 s, as follows: *Scenario A*: Based on the clinical scenario, 3D target positions extracted from projection images were used as they were (PM_CL_). *Scenario B*: The short‐arc 4D‐CBCT waveform exhibiting eight target positions was obtained by averaging the target positions in *Scenario A*. The waveform was repeated for 60 s (*W*_4D‐CBCT_) by adapting to the respiratory phase of the external surrogate. *W*_4D‐CBCT_ was used as the target positions (PM_4D‐CBCT_). *Scenario C*: The Amsterdam Shroud (AS) signal, which depicted the diaphragm motion in the superior–inferior direction was extracted from the orthogonal projection images. The amplitude and phase of *W*_4D‐CBCT_ were corrected based on the AS signal. The AS‐corrected *W*_4D‐CBCT_ was used as the target positions (PM_AS‐4D‐CBCT_). *Scenario D*: The AS signal was extracted from single projection images. Other processes were the same as in *Scenario C*. The prediction errors were calculated for the remaining 10 s.

**Results:**

The 3D prediction error within 3 mm was 77.3% for PM_4D‐CBCT_, which was 12.8% lower than that for PM_CL_. Using the diaphragm waveforms, the percentage of errors within 3 mm improved by approximately 7% to 84.0%‐85.3% for PM_AS‐4D‐CBCT_ in *Scenarios C* and *D*, respectively. Statistically significant differences were observed between the prediction errors of PM_4D‐CBCT_ and PM_AS‐4D‐CBCT_.

**Conclusion:**

PM_AS‐4D‐CBCT_ outperformed PM_4D‐CBCT_, proving the efficacy of the AS signal‐based correction. PM_AS‐4D‐CBCT_ would make it possible to predict target positions from 4D‐CBCT images without gold markers.

## 1 INTRODUCTION

Respiratory motion during radiotherapy is an issue that needs to be addressed.^1^ If large margins are added to cover the entire movement of a tumor, unnecessary doses will be delivered to surrounding healthy tissues, leading to a high possibility of radiation‐induced toxicity; therefore, motion management techniques are required for moving tumors.^2^

Techniques for respiratory motion management, such as breath‐hold techniques, respiratory gating, and real‐time tumor tracking (RTTT), have been employed in clinical practice.^3^ Among them, RTTT is ideal for the automatic and continuous field adjustment for respiratory motion.^4^

Localization of target positions is required to initiate RTTT. If the target is clearly visible on kV x‐ray images, it can be directly detected; however, the continuous kV x‐ray exposure required for monitoring of targets creates several potential issues, such as administration of a high imaging dose to patients, introduction of tracking errors caused by the sampling interval, and delays in image processing.^5^, ^6^ Targets are typically invisible on kV x‐ray images because of their small size, low density, or overlap with surrounding organs^7^; therefore, external surrogate signal‐based target localization techniques with marker implantation are widely used in clinical practice to overcome the limitations of direct target localization and conduct RTTT for such targets. This technique requires the construction of a prediction model (PM) between target positions and external surrogate signals before beam delivery, followed by subsequent prediction of the target position by inputting external surrogate signals into the PM during beam delivery. However, invasiveness, cost, and migration are issues associated with marker implantation^8^, ^9^, ^10^; therefore, because of the aforementioned issues, an external surrogate signal‐based marker‐less target localization approach is ideal.

The CyberKnife and Radixact systems (Accuray, Incorporated, Sunnyvale, CA) can realize an external surrogate signal‐based marker‐less target localization approach.^11^, ^12^ However, their target disease is limited to lung cancer, and these systems are not general‐purpose treatment machines. To popularize RTTT using external surrogate signal‐based target localization techniques, it is necessary to build a PM that uses the functions possessed by general‐purpose treatment machines.

Four‐dimensional cone‐beam CT (4D‐CBCT) is a promising option for acquiring target positions without marker implantation for PM construction. Iramina et al. demonstrated that 4D‐CBCT underestimates the amplitude of the target motion owing to low temporal resolution.^13^ If a PM is constructed from low spatiotemporal resolution information from 4D‐CBCT, the target positions might be inaccurately predicted. To the best of our knowledge, however, no researchers have evaluated the accuracy of the PM constructed using limited spatiotemporal information from 4D‐CBCT.

Furthermore, if the accuracy of the PM is not clinically acceptable, further improvement is necessary. The Amsterdam Shroud (AS) method can extract internal surrogate signals—AS signals—without marker implantation.^15^, ^16^ These signals have a high spatiotemporal resolution because they are obtained from the CBCT projection images; therefore, we hypothesize that AS signals would be beneficial in correcting the spatiotemporal uncertainties of target positions on 4D‐CBCT images. In this study, we used AS signals to develop PMs for target positions for improving the spatiotemporal resolution in target motions on 4D‐CBCT images. We then performed a comparative analysis between the PM constructed from the AS‐corrected 4D‐CBCT method that is, PM_AS‐4D‐CBCT_, and that constructed from the 4D‐CBCT method without any correction that is, PM_4D‐CBCT_.

## 2 METHODS

### 2.1 Patient characteristics and data acquisition

Twenty‐two patients with lung cancer underwent consecutive orthogonal rotational kV x‐ray imaging under free breathing using Vero4DRT (Hitachi, Ltd., Tokyo, Japan). Vero4DRT incorporates a gimbaled MV x‐ray head and an orthogonal kV x‐ray imaging system, which includes two pairs of x‐ray tubes and flat panel detectors. Notably, the imaging system rotates in sync with the gantry and O‐shaped structure. Two to four gold markers were implanted around the tumor in each patient.

The gantry rotational speed and time were 1.5°/s, and 70 s, respectively, and the image acquisition interval was 0.3°. These imaging parameters are necessary for reconstructing 4D‐CBCT with Vero4DRT. Gantry rotation angles for left or right lung cancer were 320−85° (clockwise) or 40−275° (counterclockwise), respectively. A total of 351 projection images were acquired per imager. The pixel size at the isocenter plane was 0.2 mm. During imaging, the motion of the four IR markers placed on each abdominal surface was simultaneously monitored using an IR camera with a sampling rate of 0.2 s. Details of data acquisition condition are presented in reference.^13^

The gold markers implanted around the tumor were detected in all the projection images. The 3D position of each gold marker was determined using triangulation,^17^ and the marker nearest to the tumor was selected in this study. The inclusion criterion for patients in the study was that two or three image sessions per day were successful without system interruptions; consequently, 19 patients were included in the study (Table 1). This study was approved by the Institutional Review Board of Kyoto University Hospital (approval number: 1446).

**TABLE 1:** Characteristics of patients.

Age (median [range]) (years old)	80 [65−90]
Sex (male/female)	14/5
Stage (T1a/T1b/T2a/Meta)	11/5/2/1
Location (left lung/right lung)	5/14

### 2.2 Regression‐based PM

A regression‐based PM was constructed between the IR markers and target fiducial to predict the target position during the training period.^18^ The model is mathematically expressed as follows:

(1) $$F\left(x,v\right)=ax^{2}+bx+c+dv^{2}+ev,$$

where *x* and *v* denote the centroid position and velocity of the IR markers in the anterior–posterior (AP) direction, respectively. The model parameters (*a*, *b*, *c*, *d*, and *e*) were determined by individually minimizing the residual errors between *F*(*x*,*v*) and the target position for each IR marker. Then, a single representative model was built by averaging parameters of all the models. During the tracking process, the final prediction of the target position is determined by inputting the average of all IR markers positions and velocities to the representative model.

### 2.3 Experiments

**FIGURE 1:**
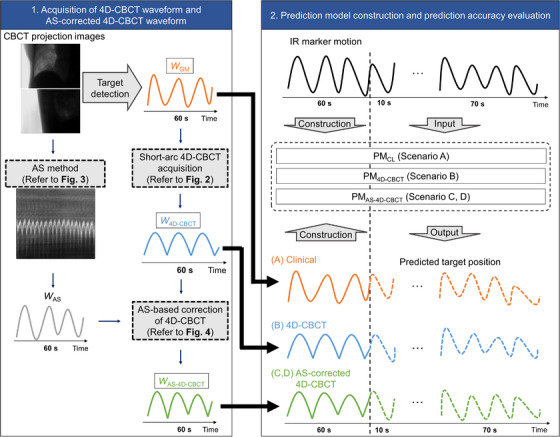
PM construction and evaluation. (Left) Procedure for creating short‐arc 4D‐CBCT waveform and AS‐corrected 4D‐CBCT waveform. (Right) PM construction and evaluation of prediction accuracy under four scenarios: (*A*) clinical, (*B*) 4D‐CBCT, (*C*) AS‐corrected 4D‐CBCT [AS was extracted from orthogonal projection images], and (*D*) AS‐corrected 4D‐CBCT [AS was extracted from single projection images]. PM, prediction model; 4D‐CBCT, four‐dimensional cone‐beam computed tomography; AS, Amsterdam Shroud; *W*_GM_, Gold marker waveform; *W*_4D‐CBCT_, Short‐arc 4D‐CBCT waveform repeated for 60 s; *W*_AS_, AS waveform; *W*_AS‐4D‐CBCT_, AS‐corrected 4D‐CBCT *W*_4D‐CBCT_; IR, infrared reflective; PM_CL_, PM constructed based on the clinical scenario; PM_4D‐CBCT_, PM constructed from IR marker waveform and *W*_4D‐CBCT_; PM_AS‐4D‐CBCT_, PM constructed from IR marker waveform and *W*_AS‐4D‐CBCT._

The following four different scenarios were considered. First, PMs were constructed using data from the first 60 s of the imaging dataset. Subsequently, the performance of these PMs was evaluated using the remaining 10 s of the imaging data, referred to as short‐term evaluation. Furthermore, the performance of the PMs constructed from the initial imaging dataset was assessed using the 70 s of the final imaging dataset from the same patient, known as long‐term evaluation. Figure 1 shows the summary of experiments conducted in this study. The details of each scenario are described below.

#### 2.3.1 Scenario A

The PM was constructed based on the clinical scenario that is, PM_CL_, wherein the IR markers’ motions and selected gold marker positions detected in all the projection images (*W*_GM_) were correlated.

#### 2.3.2 Scenario B

**FIGURE 2:**
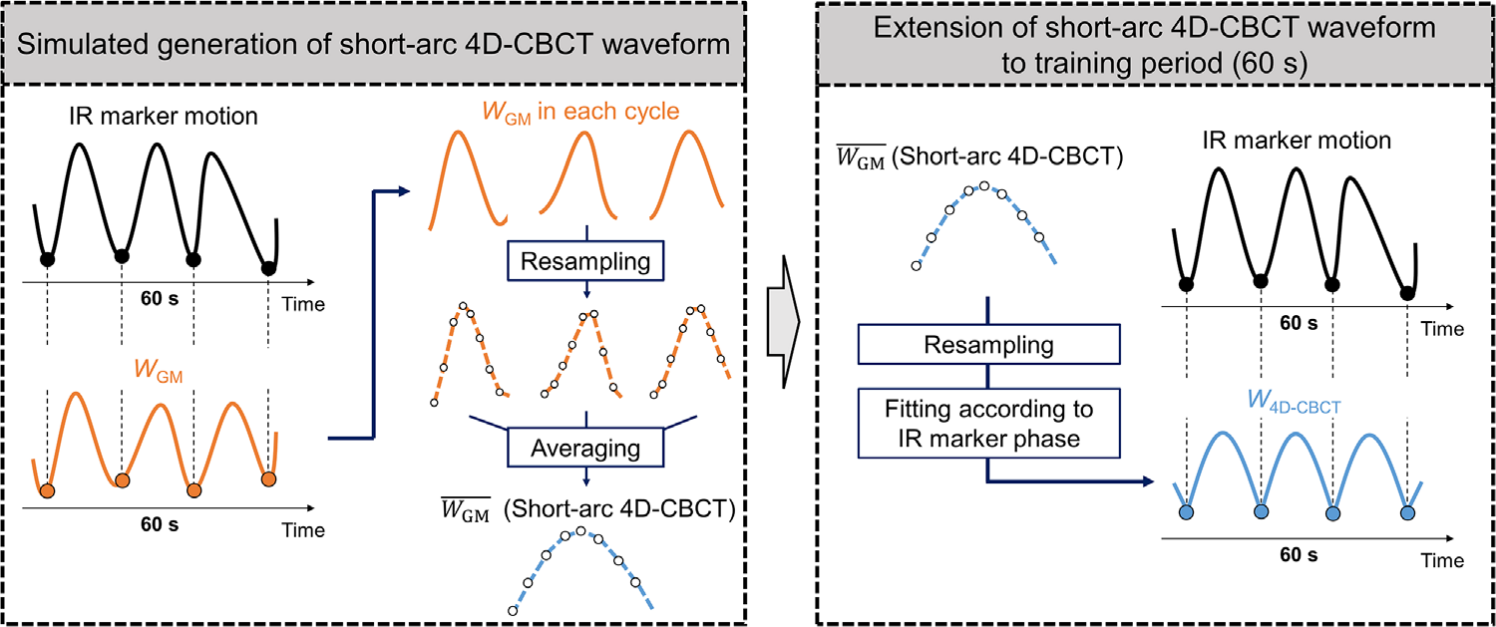
Generation of *W*_4D‐CBCT_ from *W*_GM_. $\bar{W_{\mathrm{GM}}}\;=\;$Short‐arc 4D‐CBCT waveform obtained by averaging each respiratory phase of gold marker waveform (*W*_GM_). Other abbreviations are as given in Figure 1.

The PM was constructed from the IR markers motions and gold marker positions on short‐arc 4D‐CBCT that is, PM_4D‐CBCT_. The simulated procedure for acquiring gold marker positions under short‐arc 4D‐CBCT is shown in Figure 2. As described below, we assumed that 4D‐CBCT could be reconstructed within 60 s containing *N* respirations. A total of 351 gold marker positions were divided into the first 60 s (0 ≤ *i* ≤ 301; *i*, frame number) and the remaining 10 s (302 ≤ *i* ≤ 351). Then, short‐arc 4D‐CBCT data were obtained as follows: First, *N* local minimum (LM) values, which were equivalent to the values under the end‐inhalation phase, were detected from IR signals in the range 0 ≤ *i* ≤ 301. The *N* detected frames were then indicated as (*t*_LM_[0], *t*_LM_ [1], …, *t*_LM_[*N*‐1]). Second, the *W*_GM_ was divided by the detected frames. Third, the divided *W*_GM_ was resampled into 8 respiratory phases. Finally, the values for each respiratory phase were averaged. The averaged waveform ($\bar{W_{\mathrm{GM}}}$) was regarded as a short‐arc 4D‐CBCT waveform reconstructed through phase‐based sorting.

Under $\bar{W_{\mathrm{GM}}}$, only one breathing cycle was included; therefore, $\bar{W_{\mathrm{GM}}}$ was fitted according to each detected frame using the following procedure.

- When *t*_LM_[*j*] ≤ *t* < *t*_LM_[*j*+1] (0 ≤ *j* ≤ *N*‐2), $\bar{W_{\mathrm{GM}}}$ was resampled to the *t*_LM_[*j*+1]*‐t*_LM_[*j*] frames.
- When *t* < *t*_LM_[0] or *t*_LM_[*N*‐1] ≤ *t*, $\bar{W_{\mathrm{GM}}}$ was resampled to the mean length of one respiratory cycle, *T*_mean_ $(T_{\mathrm{mean}}\;=\frac{1}{N-1}\;\sum_{j\;=\;0}^{N-2}(t_{\mathrm{LM}}[j+1]-t_{\mathrm{LM}}[j]))$. Finally, the last *t*_LM_[0] and the first 302‐*t*_LM_[*N*‐1] frames of $\bar{W_{\mathrm{GM}}}$, *t* < *t*_LM_[0] and *t*_LM_[*N*‐1] ≤ *t*, respectively, were assigned.

The obtained waveform is expressed as *W*_4D‐CBCT_.

#### 2.3.3 Scenario C

**FIGURE 3:**
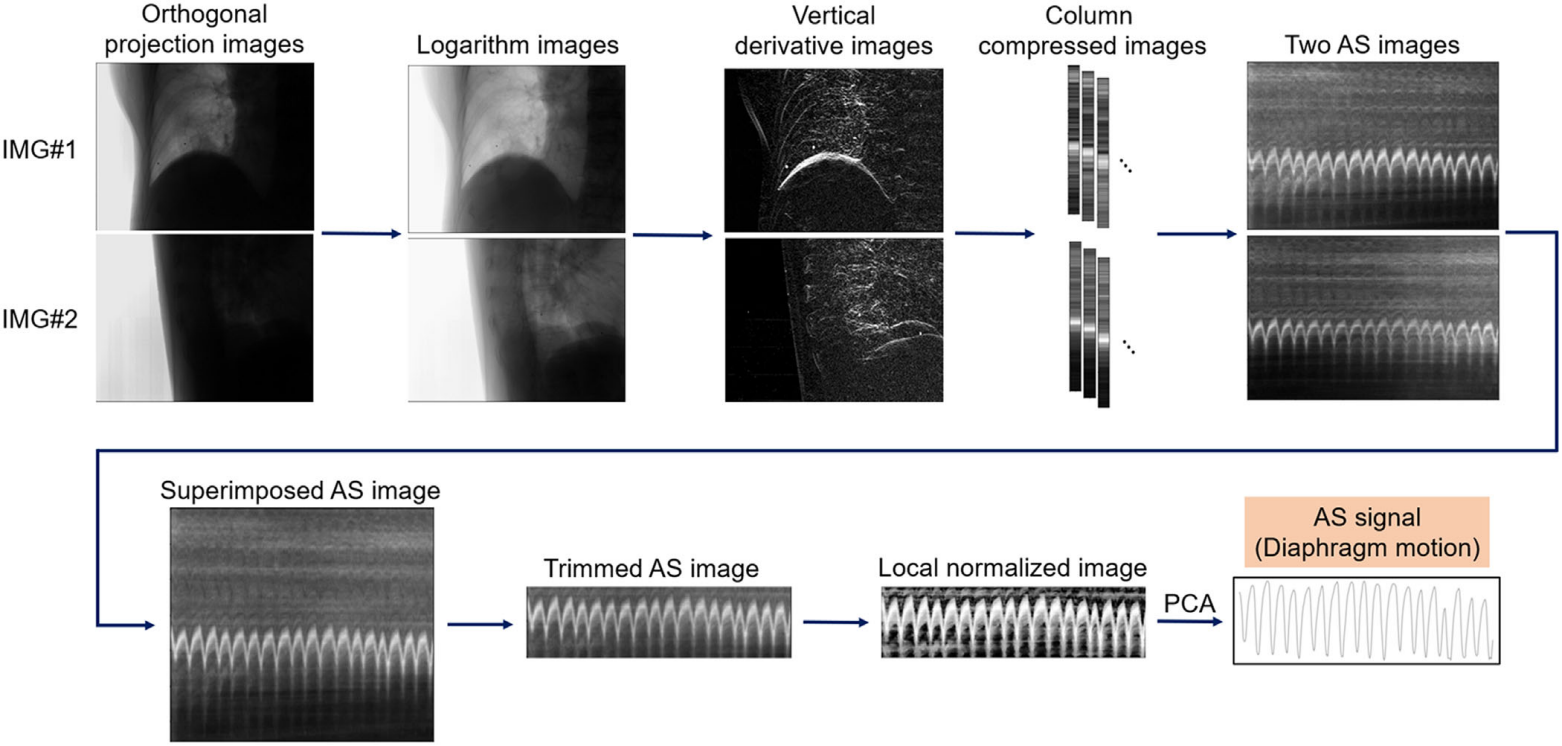
Overall workflow of AS signal extraction from orthogonal projection images. AS, Amsterdam Shroud; PCA, principal component analysis.

The PM was constructed from the IR markers and gold marker positions with the AS‐corrected 4D‐CBCT, that is, PM_AS‐4D‐CBCT_. AS‐based correction of 4D‐CBCT was done in all directions. The procedure for AS signal extraction is shown in Figure 3. First, the logarithm of each pixel value of the projection images was calculated, and a gradient along the vertical (cranial‐caudal) direction was applied. Then, each image was averaged into one column and horizontally concatenated to generate an AS image, which displays the diaphragm motion in the superior–inferior (SI) direction.^15^, ^16^ Two AS images were acquired from the pair of imagers, and they were superimposed after the diaphragm was registered in the SI direction. The superimposed image was trimmed to retain only the content around the waveform. To approximately equalize the average and variance in the pixel values of the local region, local normalization, which involves subtraction of the LM map from the original image, and subsequent local histogram equalization was applied to the trimmed image. The AS waveform (*W*_AS_) was extracted from the normalized image by performing principal component analysis (PCA) along the vertical direction.

**FIGURE 4:**
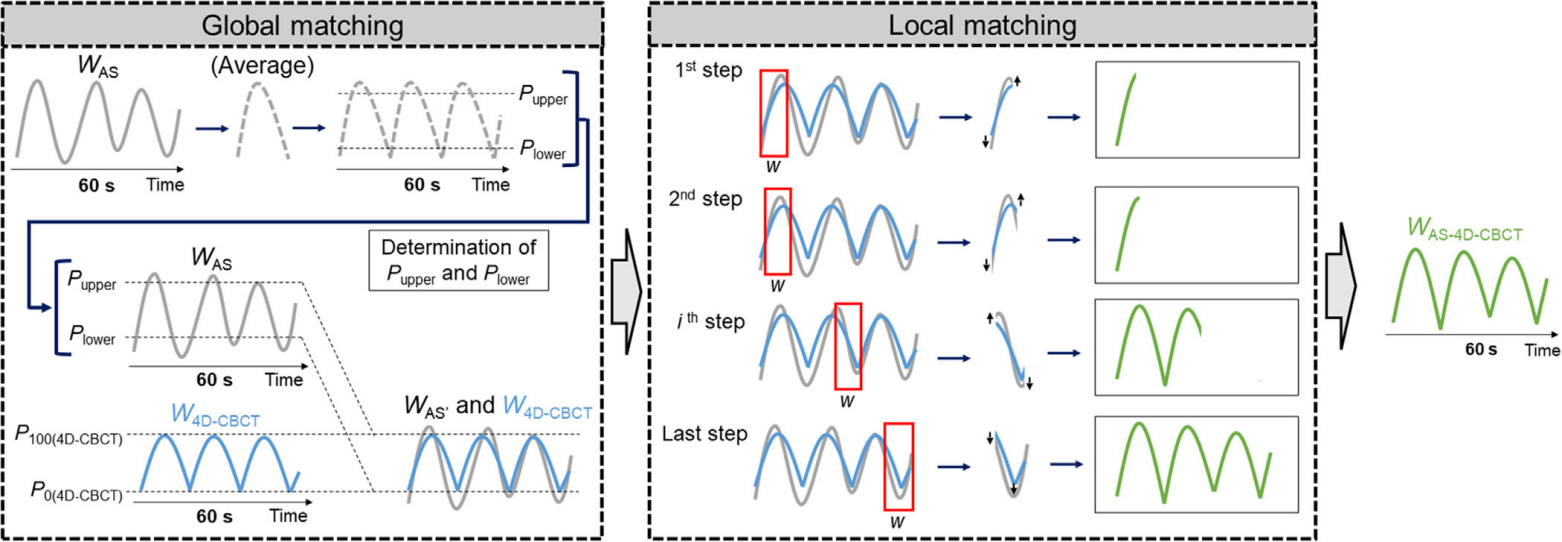
Generation of *W*_AS‐4D‐CBCT_ from *W*_AS_ and *W*_4D‐CBCT_. *W*_AS,_ AS waveform after global matching; (*P*_upper_, *P*_lower_), percentile combination used for global matching; *w*, width for local matching. Other abbreviations are as given in Figure 1.

*W*_AS‐4D‐CBCT_ was generated using *W*_AS_ and *W*_4D‐CBCT_ obtained as described in *Scenario B*. (Figure 4). This process comprises of two steps: global matching and local matching, which are described as follows.

In global matching, the motion range of the entire *W*_AS_ was matched to that of the *W*_4D‐CBCT_. Along the left–right (LR) and AP directions, the correlation between the motion of the tumor and diaphragm is often negatively correlated.^19^ This fact is also true for gold markers implanted near the tumor. If phase‐inversion was present, the phase of *W*_AS_ was inverted relative to *W*_4D‐CBCT_ by multiplying by −1 in LR and AP directions. The AS‐averaged signal was then generated using the following procedure. First, *W*_AS_ was divided by its LM map. Second, all the intervals were subjected to one‐dimensional (1D)‐spline‐interpolation so that each interval had 301 time points. Finally, all the intervals were averaged and converted to AS average signals in the same way that $\bar{W_{\mathrm{GM}}}$ was converted into *W*_4D‐CBCT_. Subsequently, the *m* ^th^ percentile height of the waveform was denoted as *P*_m_. The pair of parameters (*P*_upper,_ *P*_lower_) was defined from the AS‐averaged signal, and three different combinations, namely, (*P*_100,_ *P*_0_), (*P*_85_, *P*_10_), and (*P*_75_, *P*_15_), were selected as candidates. Global matching is mathematically expressed as follows:

(2) $$\begin{array}{ccc} W_{\mathrm{AS}}{}^{\prime } & = & \left(\frac{P_{100(4D-\mathrm{CBCT})}-P_{0(4D-\mathrm{CBCT})}}{P_{\mathrm{upper}}-P_{\mathrm{lower}}}\right)\;\left(W_{\mathrm{AS}}-P_{\mathrm{lower}}\right)\\ & & +P_{0(4D-\mathrm{CBCT}),} \end{array}$$

where *P*_100(4D‐CBCT)_ and *P*_0(4D‐CBCT)_ indicate the *P*_100_ and *P*_0_ values of *W*_4D‐CBCT_, respectively.

In local matching, the motion range of *W*_4D‐CBCT_ was locally matched to that of *W*_AS’_. To determine the number of frames in the local window *w*, 11, 21, and 31 (equivalent to 2, 4, and 6 s, respectively) windows were selected as candidates. The window was moved sequentially by a width of 1 (0.2 s). In each window, *W*_4D‐CBCT_ was shifted and scaled (linearly transformed) such that its maximum and minimum values matched those of *W*_AS’_. This procedure was repeated until the end of *W*_4D‐CBCT_. The obtained waveform is expressed as *W*_AS‐4D‐CBCT_.

#### 2.3.4 Scenario D

Our technique has the potential to be implemented in general‐purpose treatment machines capable of 4D‐CBCT imaging. In this scenario, the AS signal was extracted from single projection images because general‐purpose treatment machines commonly have single kV x‐ray imaging system. The difference from *Scenario C* was that a superimposed AS image was not used. Other processes were the same as in *Scenario C*.

### 2.4 Evaluations

First, in *Scenario C*, the parameters used for global and local matching were determined to maximize the passing rate of <3 mm 3D prediction error for PM_AS‐4D‐CBCT_. Then, the optimal parameters determined in *Scenario C* were used for AS‐based correction in *Scenario D*.

Thereafter, the accuracy of the five PMs, namely PM_CL_, PM_4D‐CBCT_, and three types of PM_AS‐4D‐CBCT_, was assessed. To clarify the difference among the three types of PM_AS‐4D‐CBCT_, the one generated in *Scenario C* was represented as ${\mathrm{PM}}_{\mathrm{AS}-4D-\mathrm{CBCT}}^{\mathrm{Dual}}$ and the others in *Scenario D* were represented as ${\mathrm{PM}}_{\mathrm{AS}-4D-\mathrm{CBCT}}^{\mathrm{Single}1}$ for imager 1 and ${\mathrm{PM}}_{\mathrm{AS}-4D-\mathrm{CBCT}}^{\mathrm{Single}2}$ for imager 2. The 3D differences between the predicted gold marker's position and actual gold marker's position were calculated. These differences were only calculated in each direction under the short‐term evaluation.

Analysis of variance (ANOVA) was performed to assess the statistical significance of the prediction errors of the five PMs. Bonferroni correction was performed for multiple tests. The level of significance was set to 0.05. The motion range of the gold marker and the averaged motion range of all IR markers were measured. The baseline drift of the IR markers between the training and testing periods was also calculated; the baseline (*BL*) was expressed as follows:

(3) $$BL=\frac{MED_{ex}\;+\;MED_{in}}{2},$$

where, *MED_ex_* and *MED_in_* indicate the median values of the averaged IR markers position in the end‐exhalation and end‐inhalation phases, respectively. A positive value indicates a baseline drift in the posterior direction.

## 3 RESULTS

Table 2 lists the characteristics of the motion of the IR and selected gold markers for the training period and the short‐term and long‐term evaluations. The median time between the two scans was 6.5 min (range: 2.5−9.0 min).

**TABLE 2:** Characteristics of implanted gold marker motion and the averaged motion of infrared reflective markers.

	Training period	Short‐term period	Long‐term period
Implanted gold marker			
Left–Right [mm]	1.7 (1.0−3.3)	1.7 (1.0−3.3)	1.7 (1.0−3.3)
Superior–inferior [mm]	11.4 (8.3−18.5)	11.6 (9.5−18.9)	11.6 (8.5−18.4)
Anterior–posterior [mm]	3.3 (2.3−5.1)	3.3 (2.2−5.5)	3.2 (2.3−5.3)
Infrared reflective marker			
Anterior–posterior [mm]	6.2 (4.0−7.8)	6.2 (4.0−7.8)	6.4 (4.3−8.2)
Breathing cycle [s]	3.7 (3.0−4.5)	3.8 (3.0−4.5)	3.8 (3.0−4.4)
Baseline drift [mm]	—	0.3 (−3.4−3.5)^a^	0.4 (−4.6−4.0)^a^

*Note*: Values are shown as median (interquartile range).

^a^ Values are shown as median (range).

Table 3 shows the 3D short‐term prediction errors within 3 mm when determining parameters used for ${\mathrm{PM}}_{\mathrm{AS}-4D-\mathrm{CBCT}}^{\mathrm{Dual}}$. The 3D prediction error within 3 mm was the highest (85.3%) with (*P*_upper_, *P*_lower_) = (*P*_85_, *P*_10_) and *w* = 21 (equivalent to 4 s). When applying the same parameters in *Scenario D*, the percentages of 3D short‐term prediction errors within 3 mm were 84.1 and 84.0% for ${\mathrm{PM}}_{\mathrm{AS}-4D-\mathrm{CBCT}}^{\mathrm{Single}1}$, and ${\mathrm{PM}}_{\mathrm{AS}-4D-\mathrm{CBCT}}^{\mathrm{Single}2}$, respectively. For comparison, the percentages were 90.1 and 77.3% for PM_CL_ and PM_4D‐CBCT_, respectively.

**TABLE 3:** Percentages of 3D short‐term prediction errors within 3 mm when determining parameters used for ${\mathrm{PM}}_{\mathrm{AS}-4D-\mathrm{CBCT}}^{\mathrm{Dual}}.$

*w* [s]	(*P*_100_, *P*_0_)	(*P*_85_, *P*_10_)	(*P*_75_, *P*_15_)
2	82.9	84.9	84.5
4	84.2	85.3	83.3
6	83.1	84.0	82.2

Abbreviations: (*P*_upper_, *P*_lower_), percentile combination used for global matching; *w*, width for local matching.

**FIGURE 5:**
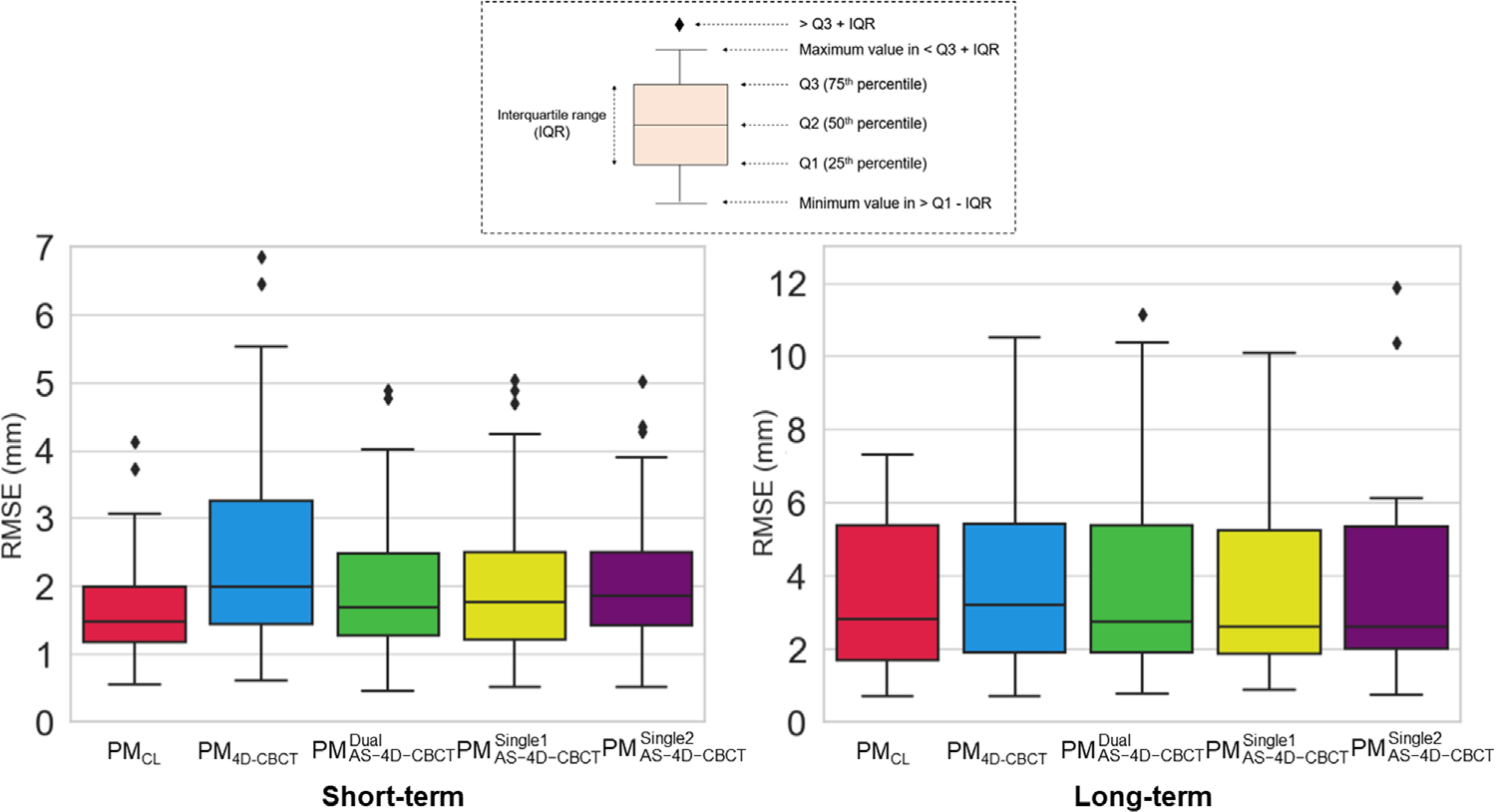
Boxplots of the root mean square errors of the 3D target prediction. (Left) short‐term evaluation and (Right) long‐term evaluation. Red, blue, green, yellow, and purple represent PM_CL_, PM_4D‐CBCT_, ${\mathrm{PM}}_{\mathrm{AS}-4D-\mathrm{CBCT}}^{\mathrm{Dual}}$, ${\mathrm{PM}}_{\mathrm{AS}-4D-\mathrm{CBCT}}^{\mathrm{Single}1}$, and ${\mathrm{PM}}_{\mathrm{AS}-4D-\mathrm{CBCT}}^{\mathrm{Single}2}$, respectively.

Figure 5 shows the boxplots of the root mean square error (RMSE). In the short‐term evaluation, the 75th percentile values were 2.0, 3.3, 2.5 mm for PM_CL_, PM_4D‐CBCT_, and all the three types of PM_AS‐4D‐CBCT_, respectively. Statistically significant differences were observed under all the approaches (*p* < 0.05); however, no statistically significant differences were observed among three types of PM_AS‐4D‐CBCT_. In contrast, in the long‐term evaluation, they ranged from 5.2 to 5.4 mm for any PMs, no statistically significant differences existed.

**FIGURE 6:**
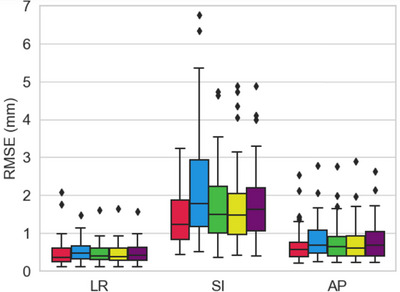
Boxplots of the root mean square errors of the target prediction in each direction. Red, blue, green, yellow, and purple represent PM_CL_, PM_4D‐CBCT_, ${\mathrm{PM}}_{\mathrm{AS}-4D-\mathrm{CBCT}}^{\mathrm{Dual}}$, ${\mathrm{PM}}_{\mathrm{AS}-4D-\mathrm{CBCT}}^{\mathrm{Single}1}$, ${\mathrm{PM}}_{\mathrm{AS}-4D-\mathrm{CBCT}}^{\mathrm{Single}2}$, respectively. AP, anterior–posterior; LR, left–right; SI, superior–inferior.

Figure 6 shows the boxplots of the RMSE in each direction for short‐term evaluation. The prediction errors were the largest in the SI direction. In the SI direction, the 75th percentile values were 1.9, 2.9, 2.2, 2.0, and 2.2 mm for PM_CL_, PM_4D‐CBCT_, and ${\mathrm{PM}}_{\mathrm{AS}-4D-\mathrm{CBCT}}^{\mathrm{Dual}}$, ${\mathrm{PM}}_{\mathrm{AS}-4D-\mathrm{CBCT}}^{\mathrm{Single}1}$, and ${\mathrm{PM}}_{\mathrm{AS}-4D-\mathrm{CBCT}}^{\mathrm{Single}2}$, respectively. Statistically significant differences were observed under all the approaches in the SI direction (*p* < 0.05); however, no statistically significant differences existed among the three types of PM_AS‐4D‐CBCT_.

**FIGURE 7:**
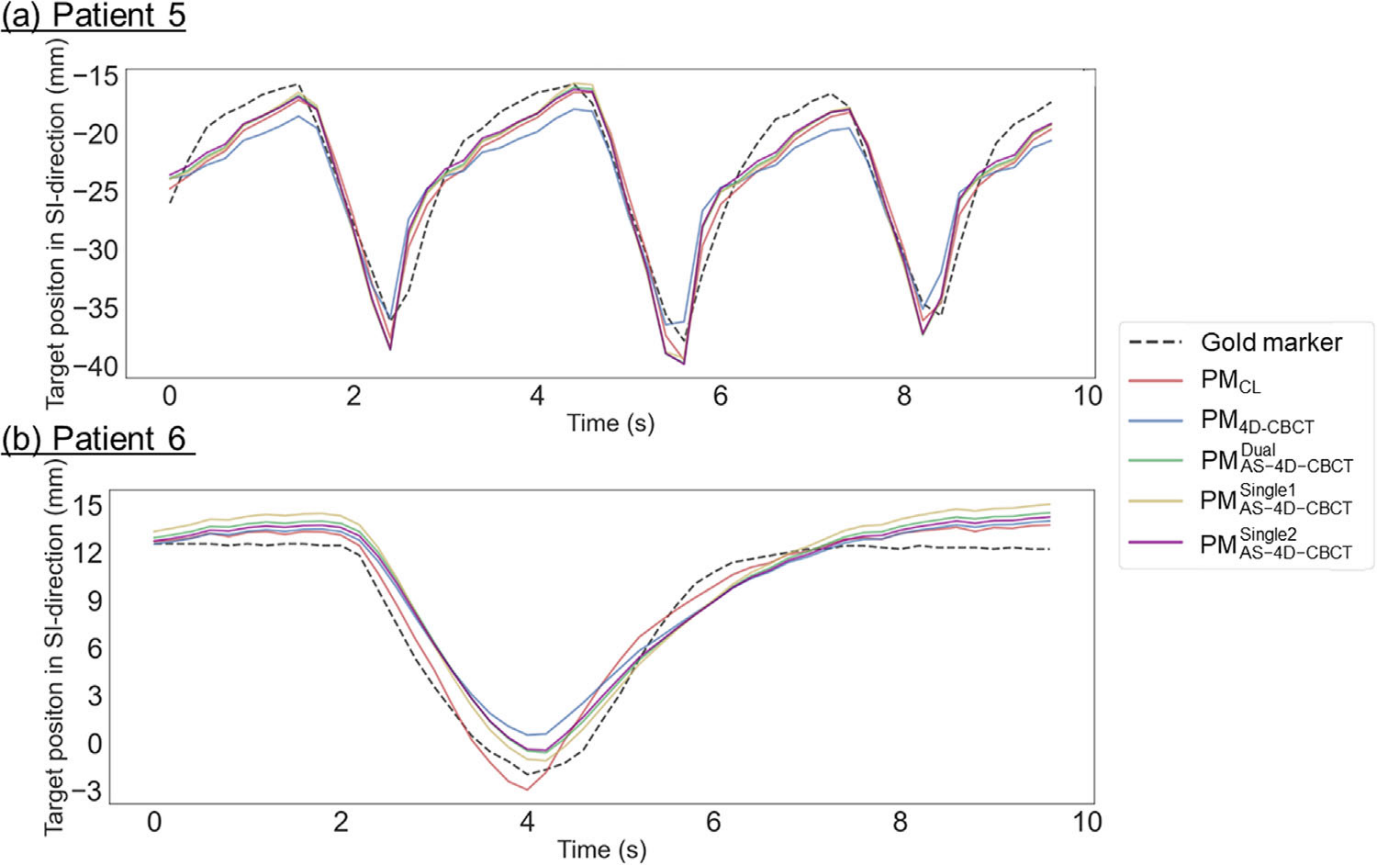
Time series of the predicted gold markers’ position in the superior–inferior direction for short‐term evaluation. Black represents the actual gold marker position. Red, blue, green, yellow, and purple represent the PM_CL_, PM_4D‐CBCT_, ${\mathrm{PM}}_{\mathrm{AS}-4D-\mathrm{CBCT}}^{\mathrm{Dual}}$, ${\mathrm{PM}}_{\mathrm{AS}-4D-\mathrm{CBCT}}^{\mathrm{Single}1}$, and ${\mathrm{PM}}_{\mathrm{AS}-4D-\mathrm{CBCT}}^{\mathrm{Single}2}$, respectively. (a) Patient 5: Performance of PM_AS‐4D‐CBCT_ was superior to that of PM_4D‐CBCT_, (b) Patient 6: Performance of PM_AS‐4D‐CBCT_ was similar to that of PM_4D‐CBCT_.

Figure 7 shows that for patients 5 and 6, the short‐term performance of three types of PM_AS‐4D‐CBCT_ was superior and similar to that of PM_4D‐CBCT_, respectively. Only the SI direction is displayed because the prediction error in the SI direction had the largest contribution to the 3D prediction error among the three directions. For patient 5, the RMSE in the short‐term evaluation in the SI direction was 1.9, 2.7, 2.0, 2.1, and 2.0 mm for PM_CL_, PM_4D‐CBCT_, ${\mathrm{PM}}_{\mathrm{AS}-4D-\mathrm{CBCT}}^{\mathrm{Dual}}$, ${\mathrm{PM}}_{\mathrm{AS}-4D-\mathrm{CBCT}}^{\mathrm{Single}1}$, and ${\mathrm{PM}}_{\mathrm{AS}-4D-\mathrm{CBCT}}^{\mathrm{Single}2}$, respectively. For patient 6, the RMSE was 1.0, 1.6, 1.6, 1.7, and 1.5 mm for PM_CL_, PM_4D‐CBCT_, ${\mathrm{PM}}_{\mathrm{AS}-4D-\mathrm{CBCT}}^{\mathrm{Dual}}$, ${\mathrm{PM}}_{\mathrm{AS}-4D-\mathrm{CBCT}}^{\mathrm{Single}1}$, and ${\mathrm{PM}}_{\mathrm{AS}-4D-\mathrm{CBCT}}^{\mathrm{Single}2}$, respectively.

## 4 DISCUSSION

In this study, we developed a PM for the target position by using diaphragm waveforms extracted from CBCT projection images. When applying the proposed method in clinical practice, first, IR marker motion monitoring and rotational x‐ray imaging are simultaneously acquired. Second, 4D‐CBCT is reconstructed, and target positions are acquired from each respiratory phase. Third, the waveform of target positions on the 4D‐CBCT is repeated for the length of the x‐ray imaging time in the same way as that described in *Scenario B*. Fourth, the repeated waveform is corrected based on the AS signal, and the PM is constructed from the IR markers signal and AS‐corrected waveform in *Scenarios C* or *D*. Finally, target localization is conducted based on the PM. While further investigation is necessary, once the tumor positions on the 4D‐CBCT are successfully detected, the proposed approach of predicting the target position using IR signals and AS‐corrected 4D‐CBCT information may have broader applicability beyond lung cancer. It could potentially be applied to low‐contrast tumors, such as liver and pancreatic cancers, without fiducial marker implantation.

To generate AS‐corrected 4D‐CBCT, the optimal parameters for global matching were determined to be (*P*_upper_, *P*_lower_) = (*P*_85_, *P*_10_). These parameters revealed that it was ideal to extend the amplitude of the AS signal than that of the short‐arc 4D‐CBCT. Global matching aims to match the motion range of the entire AS signal to that of the target; however, the low spatiotemporal resolution of the target motion in 4D‐CBCT generally results in an underestimation of the motion range^13^; therefore, the amplitude of the AS signal needs to be extended more than that of the target on the short‐arc 4D‐CBCT. The slight proximity of *P*_upper_ to *P*_50_ compared to that of *P*_lower_ is attributed to the shape of the target position waveform. Typically, the target position waveform exhibits a relatively flat pattern during the end‐exhalation phase and a steep pattern during the end‐inhalation phase. Therefore, to equally extend the upper and lower amplitudes, an asymmetry in the values of *P*_upper_ and *P*_lower_ was introduced.

The optimized parameter for local matching was determined to be *w* = 21 (equivalent to 4 s). Local matching aims to match the local maximum and LM values of the short‐arc 4D‐CBCT waveform to those of the AS signal after global matching. For *w* = 11 (equivalent to 2 s), local matching lacked stability because of an insufficient number of sampling points in the local window. On the contrary, when using *w* = 31 (equivalent to 6 s), the correction showed reduced localization due to the large window size. In conclusion, it is recommended to use a *w* value of approximately 4 s, which closely aligns with the median breathing cycle observed during the training period of 3.7 s (Table 2).

In short‐term evaluation, there were significant differences between the 3D prediction errors of PM_4D‐CBCT_ and ${\mathrm{PM}}_{\mathrm{AS}-4D-\mathrm{CBCT}}^{\mathrm{Dual}}$. The maximum difference in the prediction error between these two PMs was 2.9 mm along the SI direction; whereas, it was smaller than 0.5 mm in the LR and AP directions. These results indicate that the prediction accuracy of ${\mathrm{PM}}_{\mathrm{AS}-4D-\mathrm{CBCT}}^{\mathrm{Dual}}$ was improved compared to PM_4D‐CBCT_ because AS‐based correction compensated for the low spatiotemporal resolution in the target motions on short‐arc 4D‐CBCT, especially in the SI direction. It is generally known that the target and diaphragm motions in the SI direction are correlated to some extent.^19^ Using this fact might help enhance the prediction accuracy in the SI direction. However, the prediction accuracy in ${\mathrm{PM}}_{\mathrm{AS}-4D-\mathrm{CBCT}}^{\mathrm{Dual}}$ was significantly lower than that in PM_CL_. It implies that the diaphragm‐based correction does not perfectly reflect the movement of the target.

Although the shape of *W*_AS‐4D‐CBCT_ was slightly different among the three types of PM_AS‐4D‐CBCT_ because of the AS‐image quality, we observed no significant differences among the 3D prediction errors among them. This indicates that the accuracy of PM_AS‐4D‐CBCT_ does not statistically depend on the number of imagers or rotation angles; therefore, our proposed method is potentially applicable to general‐purpose treatment machines with a single kV x‐ray imaging subsystem.

In external surrogate signal‐based target localization, baseline drifts in the IR markers are often observed, which reduce the prediction accuracy.^20^ In clinical practice, when the baseline drift cannot be ignored, the PM needs to be updated to improve the tracking accuracy.^20^, ^21^ In this study, long‐term prediction errors were larger than short‐term errors in almost all the cases during long‐term evaluation. The baseline drift of the IR markers between the training and long‐term periods was up to 4.6 mm, which reduced the prediction accuracy of the PMs. This resulted in no significant differences among all the PMs during long‐term evaluation. In summary, a model update will be required even if PM_AS‐4DCBCT_ is used.

Our study has some limitations. First, we obtained short‐arc 4D‐CBCT by simply averaging the gold markers’ position of each respiratory phase during the first 60 s of rotational kV x‐ray imaging, although an imaging length of 70 s was required for 4D‐CBCT reconstruction using Vero4DRT. The reason for this was to verify the prediction accuracy immediately after constructing the PM. Therefore, we divided the 70 s dataset into 60 and 10 s training and testing datasets, respectively. Second, some motion artifacts are included in 4D‐CBCT images, which causes uncertainty in target positions.^13^ The development of motion artifact reduction technique is expected.^22^ Third, because clinical application of the proposed PM construction method requires imaging that lasts for 70 s to reconstruct 4D‐CBCT when using Vero4DRT, additional imaging dose will need to be delivered compared to current clinical practice wherein PMs are constructed by imaging lasting for 20 to 40 s. Nakamura et al.^5^ reported that the maximum dose administered to the 2 cm^3^ skin increased by approximately 6 cGy using the proposed method compared to that normally administered in clinical practice, which might not have a serious impact on patients.

## 5 CONCLUSION

We developed a PM construction method using AS signals to improve the accuracy of the PM constructed from target positions with low spatiotemporal resolution. The percentage of 3D prediction error within 3 mm increased from 77.3 to 85.3% with AS signal‐based correction using dual projection images. The prediction accuracy was not significantly different when using single or dual projection images. Therefore, PM_AS‐4D‐CBCT_ would make it possible to predict target positions from 4D‐CBCT images without gold markers, which can potentially help realize an RTTT approach with an external surrogate signal‐based marker‐less target localization for general‐purpose treatment machines.

## AUTHOR CONTRIBUTIONS

Y.S. and M.N. planned the study. Y.S. and S.A. performed the statistical analysis and drafted the manuscript. Y.S., S.A., and M.N. conceived the study and participated in its design and coordination. H.I., Y.I., T.M., Y.M., and T.M. helped draft the manuscript. All authors read and approved the final manuscript.

## CONFLICT OF INTEREST STATEMENT

The authors declare no conflicts of interest.

## Data Availability

The data that support the findings of this study are not available.
